# Cytotoxic T cells response with decreased CD4/CD8 ratio during mammary tumors inhibition in rats induced by non-contact electric fields

**DOI:** 10.12688/f1000research.27952.2

**Published:** 2021-05-04

**Authors:** Firman Alamsyah, Rarastoeti Pratiwi, Nisrina Firdausi, Jessica Irene Mesak Pello, Subekti Evi Dwi Nugraheni, Ahmad Ghitha Fadhlurrahman, Luthfi Nurhidayat, Warsito Purwo Taruno

**Affiliations:** 1 Center for Medical Physics and Cancer Research, Ctech Labs Edwar Technology, Tangerang, Banten, 15143, Indonesia; 2 Faculty of Biology, Universitas Gadjah Mada, Sleman, DI Yogyakarta, 55281, Indonesia

**Keywords:** non-contact electric fields, ECCT, mammary tumors, CD8+ T cells, CD4/CD8 ratio

## Abstract

**Background: **Breast cancer is the most common cancer in women worldwide and is the leading cause of death amongst women with cancer. One novel therapy used for breast cancer treatment constitutes non-contact electric fields and is called electro-capacitive cancer therapy (ECCT) with intermediate frequency and low intensity. The objective of this study was to examine the effect of ECCT on mammary tumors growth in rats and observing the immune responses that play a role in fighting the tumor.

**Methods: **Female SD rats were used and divided into four groups, namely control (NINT), placebo (NIT), non- therapy (INT), and therapy (IT) groups with 6 biological replicates in each group. Rats in INT and IT groups were treated with 7,12-dimethylbenz[a]anthracene for mammary tumor induction. Only rats in NIT and IT groups were exposed to ECCT individually for 10 hours per day for 21 days. The size of all tumors was measured with a digital caliper. The distributions of PCNA, ErbB2, caspase-3, CD68, CD4, and CD8-positive cells were observed with immunohistochemistry and scoring with ImageJ.

**Results: **The growth rate of mammary tumors in IT group was significantly lower (p<0.05) than that in INT group. The number of mitotic figures and the percentage of PCNA, caspase-3, and CD68-positive cells in IT group were significantly lower (p<0.05) than those in INT group. Conversely, the percentage of CD8-positive T cells in IT group was significantly higher (p<0.05) than that in INT group. Moreover, the CD4/CD8 ratio in IT group was found to have decreased. Some tumor tissues were blackened and detached from the surrounding tissue, resulting in an open wound which then healed upon exposure.

**Conclusions: **Non-contact electric fields exposure showed inhibition on mammary tumor growth in rats while inducing CD8+ T cells, leading to tumor cell death and potentially helping wounds heal.

## Introduction

Breast cancer is the most common cancer among women in the world, with one in eight women being at risk of developing it ^1^. Furthermore, of 18.1 million new cancer cases in 2018, around 2.1 million were breast cancer cases. This type of cancer is also the leading cause of death in women with cancer ^2^. Commonly, the treatment of breast cancer is carried out in two ways. The first, termed local therapy, controls or removes the tumor in specific areas, such as through breast-conserving therapy and mastectomy that may be followed by radiotherapy. The second, called systemic therapy, uses the circulatory system in the body to inhibit cancerous cell growth, which spread in the afflicted parts of the body. This type of treatment includes chemotherapy, targeted therapy, and endocrine therapy ^3^. However, breast cancer therapy applied so far often causes negative effects, such as the presence of pathophysiological mechanisms that cause chronic pain ^4^ or the mechanism of multidrug resistance (MDR) in breast cancer cells as a reaction to chemotherapy ^5, 6^. Moreover, breast cancer therapies may attack certain normal cells or tissues, which result in toxicity symptoms ^7^. A novel cancer therapy based on electric field exposure with intermediate frequency and low intensity called Tumor Treating Fields (TTFields) has been developed to prevent such negative effects of cancer therapy and still inhibit cancer growth. No toxic side effects or adverse events were found on animal tumor models nor patients under the exposure to this electric field-based cancer therapy, except for skin toxicity or dermatitis of low severity ^8, 9^.

Skin toxicity or dermatitis arises in electric field therapy due to prolonged direct contact between the electrodes and the skin ^10^. As such, we have developed a type of non-contact electric field therapy using capacitive modality to avoid such injuries. In our previous study in a preclinical setting, cancer therapy, namely electro-capacitive cancer therapy (ECCT), using the non-contact electric fields with intermediate frequency (100 kHz) and low intensity (18 peak-to-peak Voltage/Vpp) was found to reduce the size of breast tumors in mice significantly without causing histological damage to mammary and skin tissues. In addition, lymphocyte and macrophage infiltration was found around the tumor area through the blood vessels ^11^. Thus, one of the mechanisms of tumor cells death can be carried out with the act of anti-tumor mechanisms of various immune cells, including helper and cytotoxic T cells of lymphocytes, as well as macrophages ^12^. Cytotoxic CD8 ^+^ T cells, which are activated by helper CD4 ^+^ T cells, may enter the tumor microenvironment and induce apoptosis, resulting in shrinkage of the tumor lesions ^12, 13^. Macrophages with the M1 phenotype play an important role in the recognition and destruction of cancer cells and their presence usually indicates a favorable prognosis. On the other hand, macrophages with the M2 phenotype are involved in anti-inflammation, helping the angiogenesis process and the expression of scavenger receptors, and have a significant role in tumor development and metastasis ^12, 13^, including breast tumor ^14, 15^. One of the marker proteins to observe the infiltration of macrophages in tissues, including mammary tumor tissue, is the CD68 protein ^14– 16^.

Cancer therapy that can re-induce immune response to destroy and clean up tumor cells is a therapy that is being developed because of its positive effects. By activating immune cells, the immune cells may reach the tumor-specific areas, including the metastatic area, and specifically attack only tumor cells, not the normal cells in the vicinity ^17^. Petri *et al*. ^18^ reported that static electric fields increase the immune response in mice. Moreover, electric fields modulate the activation and polarization of some immune cells, including T cells ^19^ and macrophages ^20^. Voloshin *et al.* ^21^ reported that TTFields exposure combined with anti-PD-1 therapy induced immunogenic cell death in lung and colon tumors. In addition, Pratiwi *et al.* ^22^ reported the increased expression of CD68 and caspase-3 in rat breast tumor tissues under the exposure to 150 kHz non-contact electric fields while reducing the expression of PCNA and ErbB2 proteins. However, the study by Pratiwi *et al.* ^22^ showed an increase of the tumor size during therapy. Using a different intermediate frequency (100 kHz), we examined the effect of non-contact electric fields exposure on tumor growth in mammary tumors-induced rats and observed the activation of immune cells in fighting the tumor cells. We hypothesized that the increase in the size of mammary tumors would not occur under exposure to such non-contact electric fields with the induction of immune cells, especially lymphocytes and macrophages.

## Methods

### Ethics

This study was conducted at the Integrated Research and Testing Laboratory (LPPT) of Universitas Gadjah Mada (UGM) and at the Animal Structure and Development Laboratory of Faculty of Biology, UGM. LPPT UGM has been awarded ISO/IEC 17025:2000 accreditation for competence in testing and calibration ^22^. Experimental protocol in this study was performed following approval by the Ethical Clearance Committee of LPPT UGM with ethical clearance number: 00015/4/LPPT/IV/2017. The Ethical Clearance Committee stated that this study met the ethical requirements for research on experimental animals, and that the Ethical Clearance Committee has the right to conduct monitoring during the research.

### Experimental design

A total of 40 five-week-old healthy female Sprague Dawley (SD) rats ( *Rattus norvegicus,* Berkenhout 1769) weighing 50−80 grams were used for this study. SD rats have been used as animal tumor models to study human breast cancer with various induction agents, as well as with 7,12-dimethylbenz[a]anthracene (DMBA) administration ^23, 24^, since rats have a 98% genetic homology with humans ^25^. The rats were supplied by the LPPT UGM laboratory, Yogyakarta, Indonesia, and never used for any other experiment. Rats showing any symptoms of illness or abnormalities were excluded from the experiment. The rats were acclimated for 1 week in polypropylene cages serving as communal home cages, each of the size of 50 × 40 cm ^2^ and covered with rice hulls bedding at the bottom. One communal cage consisted of 5 rats. The temperature of the animal room was maintained at 23–26°C with 81.09% average relative humidity to avoid dehydration during electric fields exposure. Lighting condition was light from lamps during the day and total darkness during the night (12L:12D photoperiod). The rats were fed an AD2 pellet animal diet and tap water *ad libitum*. However, during treatment, cucumber slices were given as a substitute for water to avoid the animals being shocked with electricity, since the tips of the drink bottle are metal and can permit electric fields. Cage cleaning was carried out every day by cleaning the animal waste from the cage and changing food and water. Individual marking was conducted by giving picric acid to rat fur, while group marking was conducted by labelling the cage with a paint marker to avoid potential confounders.

The SD rats were divided into four groups, namely control (non-induction and non-therapy or NINT), placebo (non-induction and therapy or NIT), DMBA-induced mammary tumors without therapy (induction and non-therapy or INT), and DMBA-induced mammary tumors with therapy (induction and therapy or IT) groups. The sample size in each group was calculated according to Federer’s formula, where six biological replicates were used for each group ^22^. The animals were selected randomly and assigned to control and treatment groups. Rats were picked randomly for mammary tumors induction with a single dose of DMBA (20 mg/kg body weight), administered two times per week for five weeks. The DMBA compound is a neuroendocrine disruptor that commonly used for carcinogenesis in specific organs, including breast tumors in rats ^26, 27^. The first six rats to develop tumors were assigned to the IT group, and then six more rats were assigned to the INT group. We did this because it was technically easier to carry out the research and avoided mistakes in operating the ECCT device, since we had to turn the device on and off twice daily in the IT group, while not in the INT group. Not all rats grew tumors after DMBA administration. All healthy, normal rats in the placebo (NIT) group were given corn oil as the solvent for DMBA. All administrations were performed by the same researcher (AGF). Furthermore, each researcher in the team had different task. Besides the researcher who administered DMBA, other researchers measured the size of the nodules, recorded the morphology of mammary tumors, dissected the euthanized rats and analyzed the data statistically. One researcher (FA) controlled and monitored all the different steps in the experiment.

### Non-contact electro-capacitive cancer therapy for mammary tumors in rats

**Figure 1.**
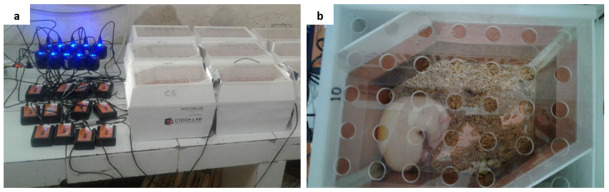
Electro-capacitive cancer therapy (ECCT) device in individual cage. ( **a**) ECCT with oscillators connected to electricity, ( **b**) a rat inside an individual cage of ECCT. The dimension of the cage was 23 cm × 18 cm × 19 cm. The arrangement of the electrodes has been described in our previous study ^11^.

Following the development of mammary tumors up to 1.5 cm in length, all rats in the IT group were exposed to intermediate frequency (100 kHz) and low intensity (18 Vpp) non-contact electric fields generated between pairs of capacitive electrodes embedded in individual cages that had been modified to an ECCT device ( Figure 1a). The device is called non-contact because its electrodes do not directly attach to the animal’s skin ( Figure 1b). All individual cages were placed on the same shelf at the same height. Non-contact electric fields therapy was performed 10 hours per day for 21 days with two hours rest after firstfive hours of therapy with the same starting time each day. The therapy was stopped when the mammary tumors had increased to 2.25 cm ^2^ in size or the last day of therapy had been reached. All rats in the placebo (NIT) group were also exposed to non-contact electric fields in the same period, three weeks after the last corn oil administration. The duration of electric fields exposure of 10 hours per day was shorter than the previous exposure in our preliminary study ^11^ to minimize any possible side effects due to exposure to electric fields ^8, 10^. After treatment, all animals were returned to their communal cages.

During therapy, all tumors were palpated once every two days and their size (cm ^2^) was measured with a digital caliper and tabulated. All nodule measurements were conducted by the same researcher (NF). In this study, we did not measure nodule size in volume due to tool limitations. The tumor size data were used to calculate the tumor surface area (TSA) using the formula for circle and ellipse areas, and subsequently, the TSA data were used to calculate the tumor growth rates (TGR) of mammary tumors. The TGR was calculated by using the specific growth rates (SGR) formula ^28^ with modification as follows: SGR (%/day) =ln (A2/A1)/(t2 –t1), where A1 and A2 are the tumor surface area before (t1) and after therapy (t2), respectively. Besides tumor size, the morphology of mammary tumors was also recorded by the same researcher (JIMP).

### Necropsy and tissue harvesting

After therapy was completed, all rats were euthanized using an overdose of ketamine (150 mg/kg of body weight) for deep anesthesia using intramuscular injection. The rats were pinned ventral side up on a dissected box and dissected by the same surgeon (AGF). Nodules and normal breast tissues were taken and washed using physiological saline. All tissues were fixed using neutral buffered formalin (NBF). Three nodules from three different rats were collected from the INT and IT groups each, while one normal breast tissue was collected from the NINT and NIT groups each. These six nodules were used for histological examination with immunohistochemical staining (IHC) of the target antigens, while the normal breast tissues were used for comparison of the overall histological structure without IHC. The number of samples used for histopathological examination with IHC staining was quite representative.

### Histological and immunohistochemical examination

Histological examination was performed by using paraffin-embedded Hematoxylin and Eosin (H&E)-stained histology slides followed by IHC ^29^ using Starr Trek Universal HRP Detection Kit Biocare. Observations and counting were performed on the mitotic figures on H&E preparations. Six antibodies purchased from Abcam Cambridge, United Kingdom, were used for IHC, namely anti-PCNA polyclonal antibody (Cat# ab18197, RRID:AB_444313), anti-ErbB2 monoclonal antibody (Cat# ab16901), anti-caspase-3 polyclonal antibody (Cat# ab13847), anti-CD68 monoclonal antibody (Cat# ab201340), anti-CD4 polyclonal antibody (Cat# ab203034), and anti-CD8 alpha monoclonal antibody (Cat# ab33786, RRID:AB_726709). The dilution for anti-CD4 and anti-CD8 antibodies was 1:750, where 1 µl of antibody was diluted with 750 µl of BSA-PBST solution (Bovine Serum Albumin 1% - Phosphate Buffered Saline with Tween® 20). On the other hand, the dilution for anti-PCNA, anti-ErbB2, anti-caspase-3, and anti-CD68 antibodies was 1:500. As much as 100 µl diluted primary antibody was applied on the appropriate slide covered with a parafilm and placed in a humidity chamber. Then, the slide was removed to a refrigerator (4°C) overnight. One to two drops (50 to 100 µl) of biotinylated secondary antibody Trekkie Universal (Biocare Medical, STUHRP700 H L10) were applied on the slides covered with a parafilm and incubated for 30 minutes at room temperature. The concentration of antibodies given was as per the manufacturer’s instructions.

### Data analysis

All measured data were analyzed with appropriate methods and without any exclusion. Semi-quantitative IHC scoring was performed using the percentage of positive area of stained cells (CD4 and CD8-positive T cells; caspase-3, ErbB2, PCNA-positive tumor cells) and the number of stained cells (CD68-positive macrophages). IHC scoring was performed in ImageJ ver.1.51, a freely available software ^22^. The comparison between positive area of stained cells and total area showed the distributions of the targeted antigens and cells ^30^. Scoring was carried out in 50 fields per group (INT and IT groups), in which 17–18 random fields were examined in one slide. In addition, CD68 ^+^ macrophages counting was performed using 60 fields per group with 20 random fields examined in a slide. In total, three slides from three different nodules were examined from each group.

All data were tested for normality (Shapiro-Wilk test, α=0.05). The data of mammary tumors growth rate from 12 animals (six rats from the IT and INT groups each) and the number of mitotic figures were analyzed using a t-test (α=0.05) to find statistical significance. The data of PCNA, ErbB2, caspase-3, CD68, CD4, and CD8-positive cells and CD4/CD8 ratio were analyzed quantitatively using non-parametric Mann-Whitney test, since the data were not normally distributed. All data were analyzed statistically using GraphPad Prism ver.8.4.3 software ^22^ for Windows by the same researcher (NF). Qualitative data (morphology of mammary tumors) were analyzed descriptively.

## Results

The outcome of this study was the growth rate of mammary tumors under exposure to non-contact electric fields. The comparison of histological characteristics between breast tissue and breast cancer in the animal tumor model, the distribution of molecular markers that described the proliferation and the mechanism of cell death, and the response of immune cells under exposure will be explained coherently in the sections below.

### The growth rate of rat mammary tumors under exposure to non-contact electric fields

**Figure 2.**
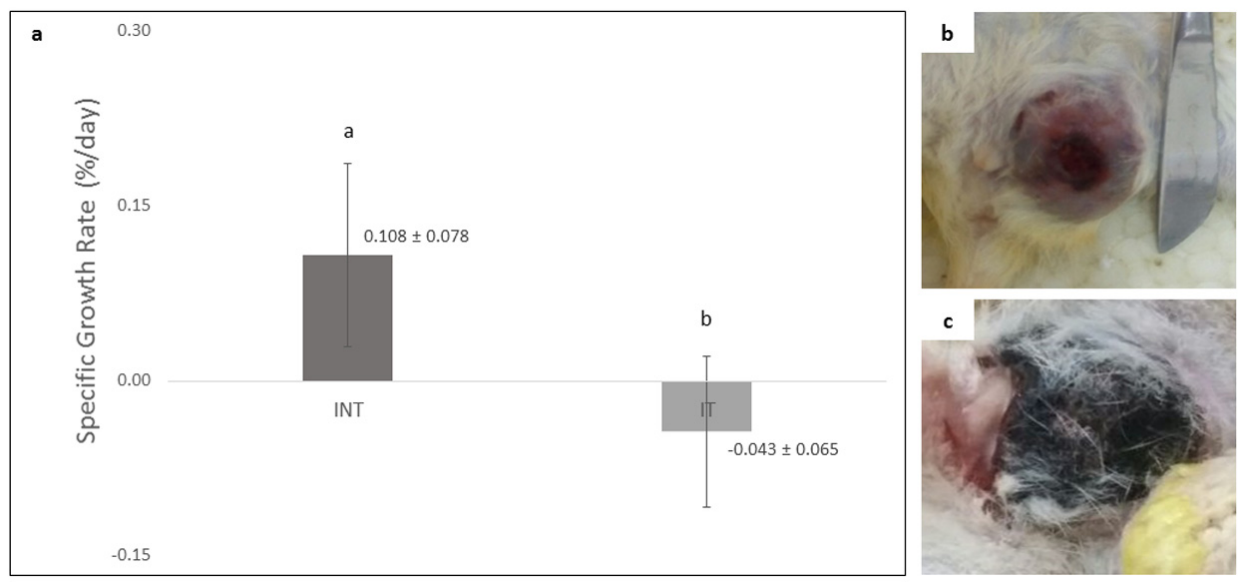
Comparison of specific growth rates (SGR) and morphology of tumors in the INT and IT groups. ( **a**) SGR of mammary tumors in the INT and IT groups, ( **b**) morphology of a mammary tumor in the INT group with the appearance of bruises, ( **c**) morphology of a mammary tumor in the IT group with a blackened condition. Different letters ( **a**, **b**) on top of the charts indicate significant differences (p<0.05).

**Figure 3.**
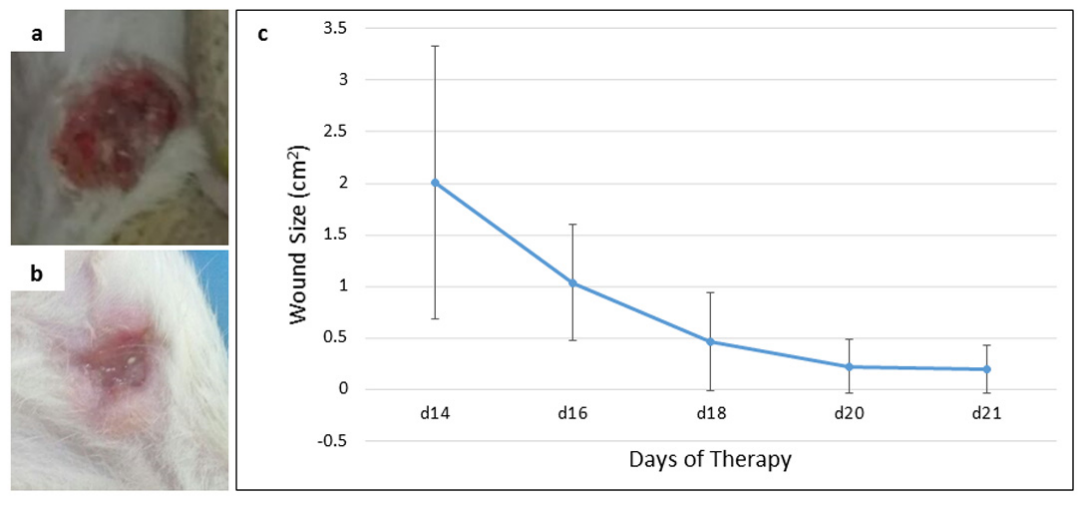
An open wound after the release of darkened tumor and its size (cm ^2^) upon exposure. ( **a**) open wound on day 14 of therapy, ( **b**) open wound on day 21 (final day) of therapy, ( **c**) the reduction of wound size from day 14 to day 21 of therapy.

The growth rate of rat mammary tumors in the IT group (-0.043 ± 0.065) was significantly lower (p<0.05) than that in the INT group (0.108 ± 0.078) and showed a negative tumor growth rate as depicted in Figure 2a. There was no tumor growth in the placebo (NIT) group. Mammary tumors morphology in INT and IT groups showed differences, in which the mammary tumors in the INT group were generally more compact, as previously reported by Pratiwi *et al.* ^22^, and appear bruised ( Figure 2b) due to thrombocytopenia from the malignancy ^31^, whereas mammary tumors in the IT group were generally softer because they contained fluid ^22^ and some were blackened ( Figure 2c). The blackened mammary tumors in the IT group were released from the surrounding tissue, as shown in Figure 3a. These tumors initially darkened, then hardened like a scab in a dry wound, and slowly sloughed off from the surrounding tissue, causing an open wound which then dried up ( Figure 3b) and shrank upon exposure to the non-contact electric field ( Figure 3c).

### Histological characteristics of breast tissue and breast cancer in rats

**Figure 4.**
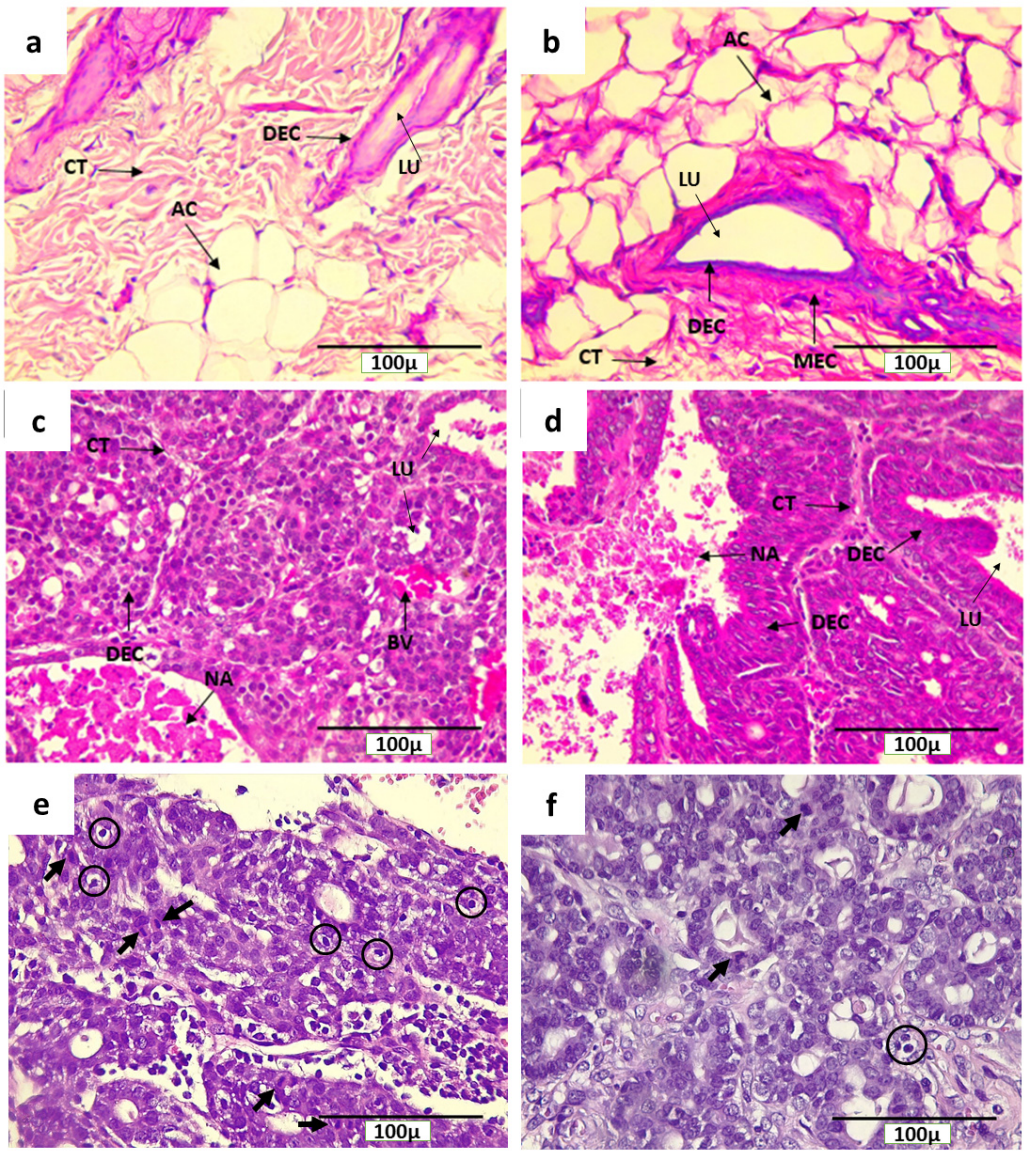
Histological features of mammary glands, mammary tumors and mitotic figures in rats with H&E staining. ( **a**) control (NINT) group, ( **b**) placebo (NIT) group, ( **c**) non-therapy (INT) group, ( **d**) therapy (IT) group, ( **e**) mitotic figures (indicated by arrows) and apoptotic bodies (in circles) in the INT group, and ( **f**) mitotic figures and apoptotic bodies in the IT group. Here, CT= connective tissue, NA= necrotic area, DEC= ductal epithelial cells, MEC= myoepithelial cells, LU= lumen, AC= adipose cells, and BV = blood vessel.

The characteristics of normal breast tissue and the pathogenesis of actively dividing cells in DMBA-induced breast cancer in rats can be seen through H&E staining ( Figure 4). Figures 4a and 4b show microscopical sections of breast tissue from rats of the NINT and NIT groups, respectively, having normal histological features with the presence of adipose tissue and other connective tissues, and in the absence of actively proliferating cells. In addition, a myoepithelial cell layer was clearly visible in the glandular epithelium ^32^. Conversely, Figures 4c and 4d show the level of malignancy of breast cancer cells in rats of the INT and IT groups, respectively. They show histological features of papillary carcinoma with the characteristics of malignant epithelial proliferation protruding into the lumen of the glandular duct, resulting in it being constricted. However, breast cancer in rats of the IT group tend to be less malignant than that in the INT group, indicated by the clear glandular duct and glandular lobule lumens as well as the prominent epithelial structure ( Figure 4d). In addition, the lumens in the IT group still maintained an intact layer of cells ( Figure 4d), while most of the lumens in the INT group did not ( Figure 4c), due to the pressure exerted by the breast cancer cells. Moreover, the region of adipose tissue and other connective tissues were also pressed by the mammary tumor cells ( Figure 4d). On the other hand, the tumor tissue itself underwent necrosis ( Figures 4c and 4d) that was characterized by inflammation ^33, 34^. The necrotic areas with inflammatory environment in the INT group were larger than those in the IT group. Figures 4e and 4f show mitotic figures and apoptotic bodies in the INT and IT groups, respectively.

### Distribution of PCNA, ErbB2, and caspase-3 in breast cancer with and without non-contact electric field exposure

**Figure 5.**
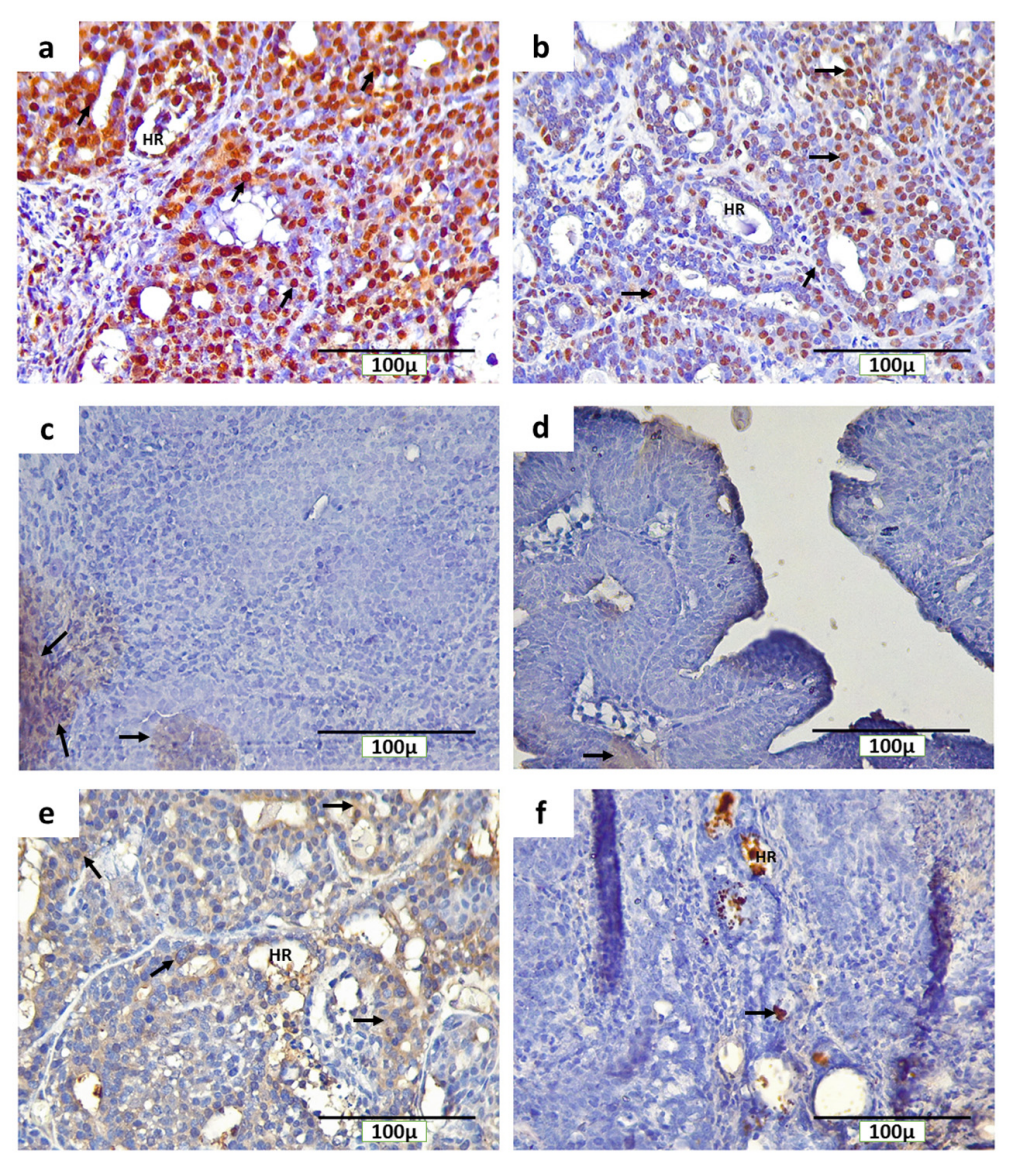
Immunohistochemical staining (3,3′-diaminobenzidine) of rat mammary tumors with PCNA, ErbB2, and caspase-3-positive cells. ( **a**) expression of PCNA in the nucleus of tumor cells in the non-therapy (INT) group, ( **b**) expression of PCNA in the nucleus of tumor cells in the therapy (IT) group, ( **c**) expression of ErbB2 on the cell membranes of tumor cells in the INT group, ( **d**) expression of ErbB2 on the cell membranes of tumor cells in the IT group, ( **e**) expression of caspase-3 in the cytoplasm of tumor cells in the INT group, ( **f**) expression of caspase-3 in the nucleus and hollow regions (HR) of tumor cells in the IT group. Brown color chromogen and arrows indicates positively stained cells.

The IHC staining with anti-PCNA, anti-ErbB2, and anti-caspase-3 detected PCNA, ErbB2, and caspase-3-positive cells, respectively, in the rat mammary tumors. The expression of PCNA in the INT and IT groups were detected in the nucleus of tumor cells ( Figures 5a and 5b, respectively), while the expression of ErbB2 in both groups was found on the cell membranes ( Figures 5c and 5d, respectively). Caspase-3 was expressed in different locations in mammary tumors of the INT and IT groups ( Figures 5e and 5f, respectively). The expression of caspase-3 in the INT group was found in the cell cytoplasm ( Figure 5e), while in the IT group, caspase-3 expression was found in the nucleus ( Figure 5f) and resulting in chromatin condensation and DNA fragmentation, indicating that apoptosis had reached a late stage ^35, 36^. In addition, the expression of caspase-3 in the IT group was also found at hollow regions in the mammary tumors ( Figure 5f), whereas caspase-3 expression in the INT group was rarely found in the same region ( Figure 5e).

**Figure 6.**
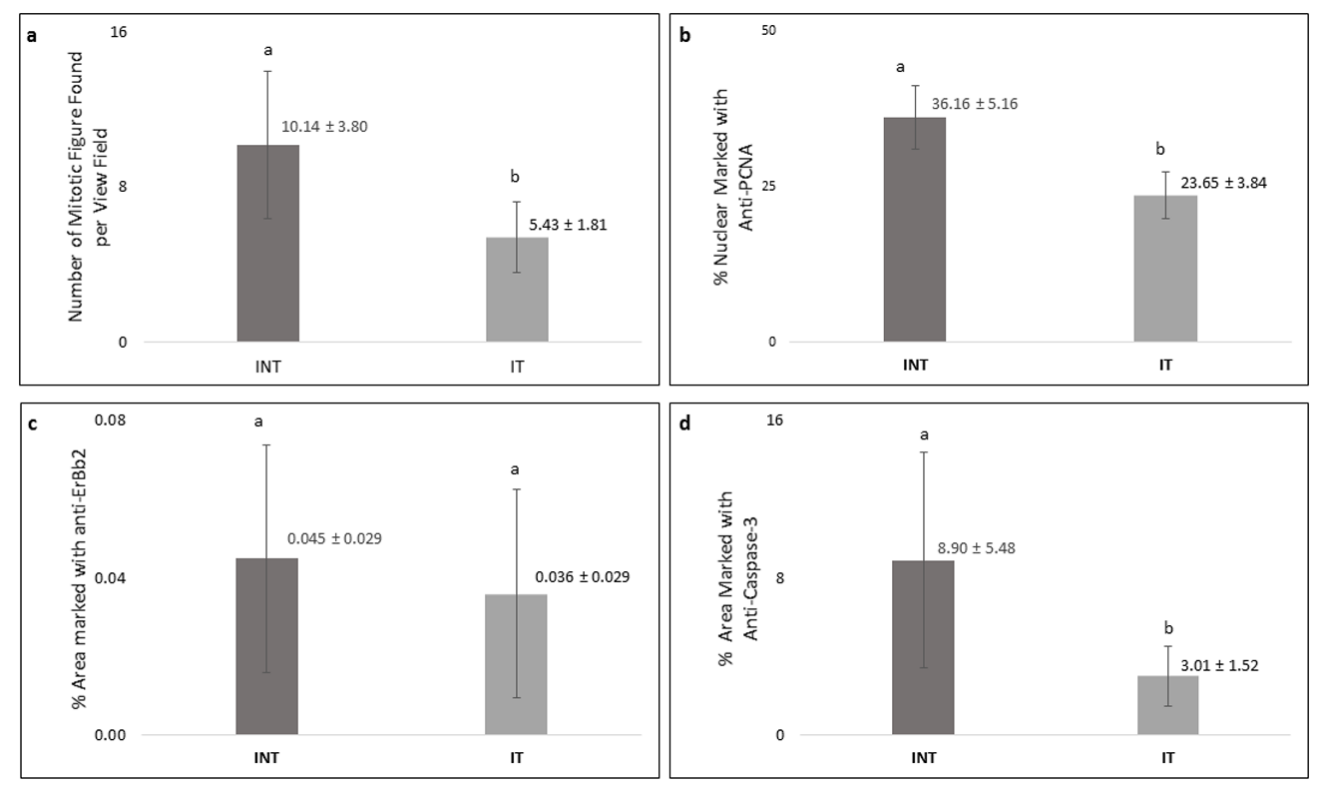
Number of mitotic figures and percentage of PCNA, ErbB2, and caspase-3-positive cells in mammary tumors. ( **a**) number of mitotic figures, ( **b**) percentage of PCNA-positive cells, ( **c**) percentage of ErbB2-positive cells, ( **d**) percentage of caspase-3-positive cells. Error bars represent the standard error of the mean. Different letters ( **a**, **b**) on top of the charts indicate significant differences (p<0.05).

Breast cancer cells that actively proliferated in rats of the INT group were seen to be relatively greater than those in the IT group, indicated by the higher number of mitotic figures per field in the INT group (10.14±3.80) as compared to that in the IT group (5.43±1.81), as shown in Figure 6a. In addition, the percentage of positively stained areas of PCNA in the INT group (36.16 ± 5.16%) was significantly higher than that in the IT group (23.65 ± 3.84%; Figure 6b). Figures 6c and 6d show that the INT group had a greater percentage of positively stained areas of ErbB2 (0.045 ± 0.029%) and caspase-3 (8.90 ± 5.48%) than those in the IT group (0.036 ± 0.029 for ErbB2 and 3.01 1.52% for caspase-3). However, the difference of percentage of positively stained areas of caspase-3 between the two groups was considered statistically significant (p<0.05), while the difference in ErbB2 expression was not significant (p>0.05).

### Distribution of CD68 ^+^ macrophages, CD4 ^+^, and CD8 ^+^ T cells in breast cancer with and without non-contact electric field exposure

**Figure 7.**
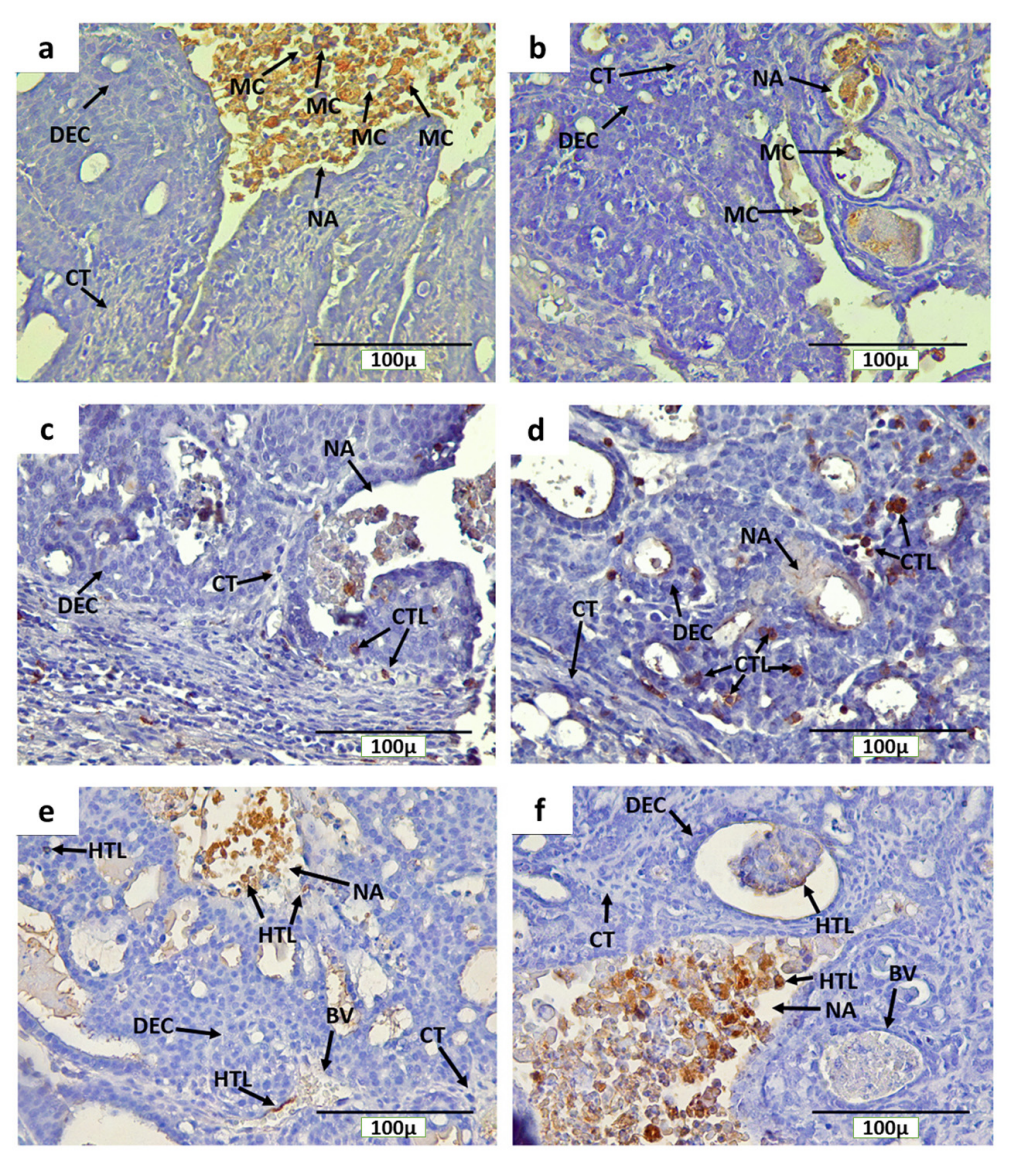
Immunohistochemical staining (DAB) of mammary tumors with CD68-positive macrophages, and CD4 and CD8-positive T cells. ( **a**) CD68 ^+^ macrophages in the non-therapy (INT) group, ( **b**) CD68 ^+^ macrophages in the therapy (IT) group, ( **c**) CD8 ^+^ T cells in the INT group, ( **d**) CD8 ^+^ T cells in the IT group, ( **e**) CD4 ^+^ T cells in the INT group, ( **f**) CD4 ^+^ T cells in the IT group. Here, brown color chromogen indicates positively stained cells. MC= macrophage cells, CT= connective tissue, BV= blood vessel, DEC= ductal epithelial cells, NA= necrotic area, CTL= cytotoxic T lymphocyte, and HTL= helper T lymphocyte.

The IHC staining with anti-CD68, anti-CD4, and anti-CD8 detected CD68 ^+^ macrophages, CD4 ^+^, and CD8 ^+^ T cells, respectively, in rat mammary tumors. The expression of CD68, CD4, and CD8 were detected on the cell membrane and in cytoplasm ( Figure 7). In addition, CD68 ^+^ macrophage infiltration was found in mammary tumors of the INT and IT groups, particularly in necrotic areas and hollow regions ( Figures 7a and 7b, respectively). The hollow regions in the IT group were seen to be more numerous and larger in size than those found in the INT group. In addition, CD68 ^+^ macrophage infiltration in the hollow regions of mammary tumors in the IT group were also seen to be greater than that in the INT group. Conversely, CD68 ^+^ macrophages infiltration in necrotic areas was seen to be higher in the INT group ( Figure 7a) than that in the IT group ( Figure 7b), since the distribution of necrotic areas in the INT group was also wider than that in the IT group. Similarly, CD4 ^+^ and CD8 ^+^ T cells were also found around the proliferating tumor cells that may respond to certain antigens in the mammary tumor cells, within blood vessels, and in the necrotic areas ^12, 13^, which were characterized by inflammation ^33, 34^ ( Figures 7c and 7d for CD8, Figures 7e and 7f for CD4).

**Figure 8.**
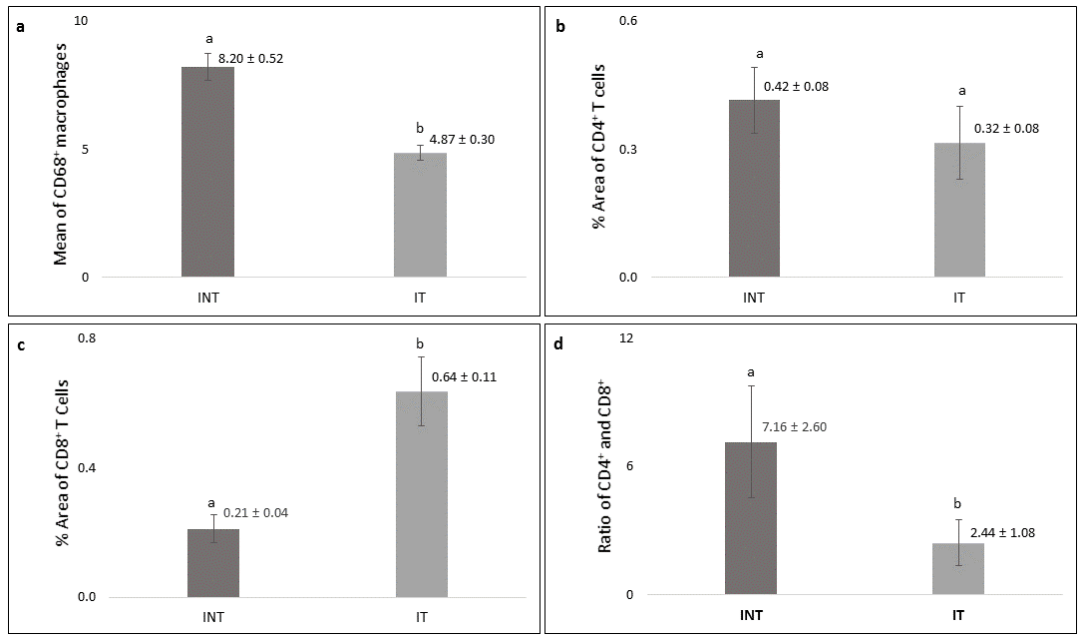
Number of CD68-positive, percentage of CD4 and CD8-positive cells, and CD4/CD8 ratio in mammary tumors. ( **a**) number of CD68-positive macrophages, ( **b**) percentage of CD4-positive T cells, ( **c**) percentage of CD8-positive T cells, ( **d**) ratio of CD4/CD8. Error bars represent the standard error of the mean. Different letters ( **a**, **b**) on top of the charts indicate significant differences (p<0.05).

A significantly higher number (p<0.05) of CD68 ^+^ macrophages was found in the INT group (8.20 ± 0.52) than that in the IT group (4.87± 0.30, Figure 8a) with a lower growth rate ( Figure 2a). The percentage of positively stained areas of CD4 in the INT group (0.42 ± 0.08%) was higher than that in the IT group (0.32 ± 0.08%), but the difference was not statistically significant (p>0.05, Figure 8b). In contrast, the percentage of positively stained areas of CD8 in the INT group (0.21 ± 0.04%) was significantly lower (p<0.05) than that in the IT group (0.64 ± 0.11%, Figure 8c). Consequently, the ratio of CD4/CD8 in the INT group (7.16 ± 2.60) was higher than that in the IT group (2.44 ± 1.08, Figure 8d).

## Discussion

In the present study, the significantly lower growth rate of rat mammary tumors in the IT group with a negative mean value of SGR as compared to the INT group with a positive mean value of SGR ( Figure 2a) showed the inhibitory effect of non-contact electric fields of ECCT against mammary tumor cells that are actively dividing in the animals, as we have previously reported in our preliminary study in mice ^11^. In addition, the development of mammary tumors exposed to 100 kHz non-contact electricfields of ECCT was decreased, unlike in the study of Pratiwi *et al.* ^22^ that used a different frequency of 150 kHz. This may suggest that differences in the frequency of the electric fields can give different therapeutic results in cancer treatment ^37, 38^ and that the 100 kHz was the appropriate frequency of non-contact electric fields in inhibiting the proliferation of mammary tumor cells. These results are consistent with the results of our *in vitro* studies using various frequencies (100 kHz, 150 kHz, and 200 kHz) and intensities (10 Vpp, 18 Vpp, and 30 Vpp) to inhibit breast cancer cell proliferation, and it was found that a frequency of 100 kHz and an intensity of 18 Vpp were the optimum electric fields exposure to inhibit breast cancer cell proliferation ^11^.

There are several deviation values that are quite large in some data that show quite different responses from each individual rat to the electric field exposure. For example, in the SGR data ( Figure 2a), there is indeed 1 outlier data in the IT and INT groups each. These can occur because the estrous cycle that stimulates prolactin can be different in each individual mouse, which can affect differences in the growth rate of mammary tumors in rat ^39, 40^. In addition, different levels of stress due to therapy in individual cages could affect differences in the development of mammary tumors in individual rat ^41, 42^.

The absence of tumors in the placebo (NIT) group ( Figure 4b) demonstrated the safety of non-contact electric fields of ECCT to healthy or normal animal models, as we have previously reported ^11^. The safety of electric field exposure in normal tissue is determined not only by the frequency and intensity used ^37^, but also by the membrane potential of the normal cell, which is higher than cancer cell ^43, 44^. External electric field exposure cannot penetrate normal cell membranes due to the higher membrane potential ^37, 43, 44^ and only causes rotational motion of the cell ^37^. On the other hand, external electric field exposure can penetrate cancer cell membranes with lower membrane potential, thereby interfering with mitosis ^37, 43, 44^. However, not all normal cells have a higher membrane potential than cancer cells. For example, liver cell has the same membrane potential as MCF-7 breast cancer cell ^43, 44^. Even so, we found that liver structure and function were not impaired under exposure to ECCT’s electric fields based on histological examination, and alanine transferase activity and bilirubin levels in blood plasma, respectively. The data of the safety of ECCT exposure in rat, particularly in the structure and function of liver and kidney, and haematological profiles, will be published separately.

In addition to its safety, this exposure did not produce acute damage to rat skin tissue or chronic pain that usually occurs after radiation therapy, including after breast cancer radiation therapy ^4, 45, 46^. Instead, it helped wound healing after cancer treatment in rats of the IT group, indicated by the drying wound and the reduction of wound size ( Figures 3b and 3c). This shows that endogenous electric fields are known to play the role of a signal that directs cell migration in epithelial wound healing. Thus, external electric fields exposure may accelerate wound healing by directing the migration of T cells ^19^ and fibroblast, as well as their proliferation and transdifferentiation ^47, 48^. In addition, Hoare *et al*. ^20^ reported that external electric fields may contribute to the coordination and regulation of macrophage functions, including wound healing. The effect of non-contact electric fields of ECCT in directing immune cells was also seen in the fluid contained in the mammary tumors of the IT group, as indicated by the presence of macrophages and lymphocytes infiltration in the tumor area ( Figure 7) as we have previously reported ^11, 22^. This immune cell infiltration can promote the destruction of cancer cells ^12, 21, 49^, as demonstrated in the IT group ( Figures 2a and 2c).

The cellular effects of electric fields exposure on PCNA expression during the S-phase may indicate the incidence and the growth rate of mammary tumors ^50^. Since PCNA is an S-phase marker in the interphase that has a central role in DNA replication and repair ^51^, the decrease of PCNA expression in mammary tumor cells in the IT group ( Figure 6b) may indicate a decrease in the incidence and the growth rate of mammary tumors ^50^. In addition, alternating electric fields exposure with intermediate frequency and low intensity used in this study can cause tumor cells that are dividing to experience uneven segregation of chromosomes and mitotic catastrophe, followed by programmed cell death ^52^. This is supported by the study of Mujib *et al*. ^53^, which reported that the p53 protein can serve as a marker for cancer cell death via apoptosis due to non-contact electric fields exposure. Moreover, the hollow cavities with cell debris, also called dead cell islands, may be the spaces in which mammary tumor cells used to be present and then died in through apoptosis ^54, 55^, since caspase-3 was found here in the IT group ( Figure 5f). In addition, within these dead cell islands, the expression level of caspase-3 was very low ^55^, as found in the IT group ( Figure 6d).

The lower expression of caspase-3 in the IT group ( Figure 6d) may suggest the mechanism of mammary tumor cell death under exposure to non-contact electric fields of ECCT through caspase-independent cell death. Meggyeshaz *et al.* ^56^ reported tumor destruction by electric fields exposure and concomitant heat with elevated DNA fragmentation and low levels of caspase-3 expression localized to inflammatory cells, which dominantly follows a caspase-independent pathway. Meanwhile, higher caspase-3 expression in the INT group ( Figure 6d) suggested that higher apoptosis events may have occurred in this group as a result of oxidative stress due to elevated reactive oxygen species in mammary tumors ^57, 58^. Although apoptosis occurred in the mammary tumors, the tumor cell death rate was relatively much lower than the proliferation rate, so mammary tumor size increased ^59^, as seen in the INT group ( Figures 2a and 2b).

The type of receptor possessed by breast cancer cells in this study was the ErbB2 receptor ( Figures 5c and 5d). The generation of mammary tumors with ErbB2 receptors was the result of carcinogenesis due to DMBA administration into the animal tumor model ^60^. Despite such insignificant results in the expression level of ErbB2 between INT and IT groups ( Figure 6c), tumor size in the INT group continued to increase from the baseline, and the tumor growth rate was higher than that in the IT group ( Figure 2a). In addition, the number of mitotic figures and the percentage of PCNA-positive tumor cells in the INT group were also higher than those in the IT group ( Figures 6a and 6b, respectively). This suggests that the INT group experienced higher tumor cell proliferation than the IT group. Moreover, the higher number of CD68 ^+^ macrophages as one of inflammatory cells seen in the tumor microenvironment of the INT group ( Figure 7a and Figure 8a) was capable of inducing -tumor growth by contributing to cellular migration of tumor cells and metastasis ^12, 14, 61^. Ong *et al.* ^62^ also reported that rat mammary carcinoma had a significantly higher percentage of CD68 ^+^ macrophages as compared to benign mammary tumors with a lower growth rate. CD68 ^+^ macrophages that infiltrate the necrotic areas of the INT group ( Figure 7a) may be considered as M2 macrophages which play a role in tumor growth and provide a poor prognosis ^12, 62, 63^. In addition, increased M2 macrophage or tumor-associated macrophage (TAM) infiltration in mammary tumors would be accompanied by an increase in tumor size ^14, 15, 64^, as shown in the INT group ( Figure 2a and Figure 8a). Conversely, the lower the number of infiltrating TAMs in rat mammary tumors, the smaller the size of the tumors ^12, 15^, as shown in the IT group ( Figure 2a and Figure 8a). Moreover, the expressions of chemokine (C-C motif) ligand 2 (CCL2) and interleukin-18 (IL18) that have main roles in the development of metastatic breast cancer and excessive angiogenesis by amplifying M2 macrophage polarization ^65, 66^ were downregulated under the exposure to non-contact electric fields of ECCT at 150 kHz ^22^. Similarly, non-contact electric field (100 kHz) exposure in our study may also inhibit the amplification of M2 macrophage polarization, resulting in the negative mammary tumor growth rate in rats of the IT group ( Figure 2a). The infiltration of CD68 ^+^ macrophages in mammary tumors of the IT group suggested that exposure to non-contact electric fields of ECCT may direct macrophages, likely of the M1 phenotype, to the tumor areas ^11, 21^ and then engulf and digest dead cell, debris and tumor cells ^12, 67^ that eventually produced hollow cavities ^54, 55^ as seen in Figure 7b. This macrophage-mediated clearance of dead cells occurred as caspase-3-independent death ^68^, since the IT group showed a lower expression of caspase-3 than that in the INT group ( Figure 6d).

Immunogenic tumor cell death induced by the electric fields exposure in the IT group can also be caused by the interaction of lymphocyte with receptors on the membrane of mammary tumor cells with the production of cytokines ^21, 52^. The distribution of CD4 ^+^ and CD8 ^+^ T cells within blood vessels and mammary tumors of the IT group revealed that non-contact electric fields of ECCT may direct the spreading of these lymphocytes from blood vessels to tumor areas ^11, 19, 21^, including necrotic areas in it ( Figures 7d and 7f). The presence of CD4 ^+^ T cells in mammary tumors would induce the activation of CD8 ^+^ T cells to kill the tumors ^12, 69, 70^ as an adaptive immune response ^12, 13, 71^. However, since the percentage of CD4 ^+^ T cells in the INT and IT groups was not significantly different ( Figure 8b), the presence of CD4 ^+^ T cells in the tumor areas may not always indicate tumor elimination; instead, they may indicate tumor progression ^12, 30, 72^. Moreover, electric field exposure can decrease CD4 ^+^ T cells polarization ^19^, resulting in the lower percentage of CD4 ^+^ T cells in the IT group as compared to the INT group ( Figure 8b). Conversely, the presence of CD8 ^+^ T cells in the necrotic areas, which is characterized by inflammation ^33, 34^, indicated their role in the inflammatory response to clear dead cells ^12, 73^ recognized as foreign agents ^13^. In addition, a higher percentage of CD8 ^+^ T cells in the IT group ( Figure 8c) indicated an increased anti-tumor immunity and immunological ability against tumor progression ^72, 74, 75^. CD8 ^+^ T cells may release granzymes during granule exocytosis when tumor cells are marked for elimination ^12, 13, 76^, and the granzymes may induce caspase-independent apoptotic pathways ^77, 78^ which support the presence of hollow cavities in the tumor areas with low caspase-3 expression ^55^ as seen in the IT group ( Figure 5f and Figure 6d).

The ratio of CD4/CD8 is correlated to clinical aspects and could be used as prognostic factor for breast cancer, where a higher CD4/CD8 ratio in the INT group ( Figure 8d) correlated to mammary tumor progression ^79^. Conversely, the higher positive area of CD8 ^+^ T cells and the lower ratio of CD4/CD8 in the IT group suggested a good prognosis in rat breast cancer ^12, 72, 79^ with non-contact electric field exposure. Further study will be conducted to investigate the potency of non-contact electric fields exposure in wound healing upon breast cancer treatment by observing the presence of cytokines, as well as the migration of immune cells and fibroblast that regulate wound healing ^20, 47, 48^ by using tissue samples from the IT group. Based on the evidence of its efficacy and safety on preclinical studies, the 100 kHz non-contact electric field has been used in phase I clinical trials of ECCT for healthy volunteers. The data of the safety of ECCT exposure in healthy volunteers will be published separately.

## Conclusions

In summary, low intensity (18 Vpp) and intermediate frequency (100 kHz) non-contact electric fields exposure showed inhibition of mammary tumor growth in rats by inducing CD8 ^+^ T cells that lead to tumor cell death supported by a decreased CD4/CD8 ratio. It also showed inhibition of M2 macrophage polarization, resulting in the negative tumor growth rate. The proposed therapy gives good prognosis in rats with mammary tumors and potentially helps in wound healing of the tumor after the treatment.

## Data availability

### Underlying data

Open Science Framework: Cytotoxic T cells response with decreased CD4/CD8 ratio during mammary tumors inhibition in rats induced by non-contact electric fields, https://doi.org/10.17605/OSF.IO/3P794 ^80^ (registered on 11 December 2020: https://osf.io/b6d4m).

This project contains the following underlying data:

- Ethical clearance document

- Growth rate of mammary tumors and wound healing data

- IHC scoring data

- Raw images for IHC and hematoxylin figures

- Statistical analysis with GraphPad Prism ver.8.4.3

Data are available under the terms of the Creative Commons Zero “No rights reserved” data waiver (CC0 1.0 Public Domain Dedication).
