# Supplementary material for: Cytotoxic T cells response with decreased CD4/CD8 ratio during mammary tumors inhibition in rats induced by non-contact electric fields
Source: F1000Res. 2021 May 4;10:35. Originally published 2021 Jan 19. [Version 2] doi: 10.12688/f1000research.27952.2 (PMC8142601; doi:10.12688/f1000research.27952.2)
Supplement: Peer review history

# Peer review: Reviewer response for version 2

**DOI:** 10.5256/f1000research.55837.r84419

**Published:** 2021-05-21

**Authors:** Bilgin Keserci

**Affiliations:** 1 School of Medical Sciences, Universiti Sains Malaysia, Kelantan, Malaysia

The authors responded to my concerns well. I do not have any further questions. I strongly recommend indexing.

Is the work clearly and accurately presented and does it cite the current literature?

Yes

If applicable, is the statistical analysis and its interpretation appropriate?

Yes

Are all the source data underlying the results available to ensure full reproducibility?

Yes

Is the study design appropriate and is the work technically sound?

Yes

Are the conclusions drawn adequately supported by the results?

Yes

Are sufficient details of methods and analysis provided to allow replication by others?

Yes

Reviewer Expertise:

Medical imaging, cancer therapy, MRI, image guided therapies

I confirm that I have read this submission and believe that I have an appropriate level of expertise to confirm that it is of an acceptable scientific standard.

# Peer review: Reviewer response for version 1

**DOI:** 10.5256/f1000research.30917.r79324

**Published:** 2021-03-03

**Authors:** Elfahmi Elfahmi

**Affiliations:** 1 School of Pharmacy, Bandung Institute of Technology, Bandung, Indonesia

The article consists a quite good scientific background and methodology and has an innovation to be contributed in the treatment of cancer, however other clinical studies should be conducted. The quality of works and article is suitable for indexing, however, some comments should be addressed by author for the statement or question as follow:

1. Some data presented in the results showed a quite big deviation, are there any outliers in the 6 replicates data which contributed for this deviation? If it’s possible that the authors could explain this clearly, it would then strengthen the paper.
2. Although the electrical field is not contacted with the animal models, it is placed in the closed chamber. Could the authors explain in scientific view whether there are no side effects of the electric field?
3. The condition parameter of electric field used in this study is only point. Is it an optimum condition? If yes, the proven study for the optimalization should be included.
4. If there is toxicity data of the instrument published, it would be better if included.

Is the work clearly and accurately presented and does it cite the current literature?

Yes

If applicable, is the statistical analysis and its interpretation appropriate?

Yes

Are all the source data underlying the results available to ensure full reproducibility?

Yes

Is the study design appropriate and is the work technically sound?

Yes

Are the conclusions drawn adequately supported by the results?

Yes

Are sufficient details of methods and analysis provided to allow replication by others?

Yes

Reviewer Expertise:

NA

I confirm that I have read this submission and believe that I have an appropriate level of expertise to confirm that it is of an acceptable scientific standard.

# Response

**Published:** 2021-03-30

**Authors:** Firman Alamsyah

**Affiliations:** Ctech Labs Edwar Technology, Indonesia

1. The deviation value that is quite large in some data shows a quite different response from each individual rat to the electric field exposure. For example, in the SGR data (Figure 2), there is indeed 1 outlier data in each of the IN and INT groups. This may occur because the estrous cycle that stimulates prolactin can be different in each individual mouse which can affect differences in the growth rate of mammary tumors in rat (Nandi et al., 1995; Schedin et al., 2000). In addition, different levels of stress due to therapy in individual cages could affect differences in the development of mammary tumors in individual rat (Hermes et al., 2009; De la Roca-Chiapas, et al., 2016). We have added this argument in the revision of our article.

 2. We have explained the safety of electric field of ECCT to mammry and skin tissues of animal models in the second paragraph of the Introduction as reported in our previous publication. In addition, ECCT has passed the phase I clinical trials for healthy volunteers as stated in the last sentence of the Discussion. The safety of ECCT in normal tissue is determined by the membrane potential of the tissue which is higher than that in cancer cells (Yang and Brackenburry, 2013; Kadir et al., 2018). External electric field exposure cannot penetrate normal cell membranes due to the higher membrane potential (Kirson et al., 2004; Yang and Brackenburry, 2013; Kadir et al., 2018) and only causes rotational motion of the cell organelles (Kirson et al., 2004). On the other hand, external electric field exposure can penetrate cancer cell membranes with lower cell membrane potential, thereby interfering with mitosis (Kirson et al., 2004; Yang and Brackenburry, 2013; Kadir et al., 2018). However, not all normal cells have a higher membrane potential than cancer cells. For example, liver cell has the same membrane potential as MCF-7 cancer cell (Yang and Brackenburry, 2013; Kadir et al., 2018).  Even so, we found that liver function was not impaired based on alanine transferase activity and bilirubin levels in blood plasma. The data of the safety of ECCT exposure in rat and healthy volunteers will be published separately this year.

 3. We have tried various frequencies (100, 150, and 200 kHz) and intensities (10 Vpp, 18 Vpp and 30 Vpp) of the ECCT electric field in preclinical studies and found that a frequency of 100 kHz and an intensity of 18 Vpp are the optimum electric field exposure for breast cancer therapy.

 4. ECCT toxicity data on the liver, kidney and hematological profiles will be published separately this year.

# Peer review: Reviewer response for version 1

**DOI:** 10.5256/f1000research.30917.r79321

**Published:** 2021-02-23

**Authors:** Bilgin Keserci

**Affiliations:** 1 School of Medical Sciences, Universiti Sains Malaysia, Kelantan, Malaysia

Even though the study results are quite impressive, I have some additional issues to be considered/corrected before this paper is indexed.

 First, the manuscript needs serious language editing. I strongly advise them to get a professional editing service when revising. 

 Second, the below concerns must be answered and explained well in the manuscript before being indexed:

 On page 4 it says, *"The first 6 rats to develop tumors were assigned to IT group, and then 6 more rats were assigned to INT group. Not all rats grew tumors after DMBA administration. All healthy, normal rats in the placebo (NIT) group were given corn oil as DMBA (7,12-dimethylbenz[a]anthracene) solvent."*

**Questions:**

- What does this mean? How many rats developed tumors in the IT and INT groups? Why were all the rats that developed the tumor first included in the IT group rather than equally distributed to INT and IT?
- Were the rats in the NIT group taken from the non tumor growing induced group after being given corn oil?

On page 5 it says, *"Three nodules from three different rats were collected from each INT and IT groups, while one normal breast tissue was collected from each NINT and NIT groups."*

**Question:**

- Why not all? How are the rats chosen?

On page 7 it says, *"In addition, the percentage of positively stained areas of PCNA in INT group (36.16 ± 5.16%) was significantly higher than that in **INT** group (23.65 ± 3.84%, Figure 6b)."*

**Comment:**

- 'INT' in bold above is supposed to be 'IT' group. Please correct it.

Is the work clearly and accurately presented and does it cite the current literature?

Yes

If applicable, is the statistical analysis and its interpretation appropriate?

Yes

Are all the source data underlying the results available to ensure full reproducibility?

Yes

Is the study design appropriate and is the work technically sound?

Yes

Are the conclusions drawn adequately supported by the results?

Yes

Are sufficient details of methods and analysis provided to allow replication by others?

Yes

Reviewer Expertise:

Medical imaging, cancer therapy, MRI, image guided therapies

I confirm that I have read this submission and believe that I have an appropriate level of expertise to confirm that it is of an acceptable scientific standard, however I have significant reservations, as outlined above.

# Response

**Published:** 2021-02-27

**Authors:** Firman Alamsyah

**Affiliations:** Ctech Labs Edwar Technology, Indonesia

1. We will consider getting a professional language editing service to revise our manuscript.

 2. The rats were randomly selected for mammary tumor induction with DMBA administration. One month after DMBA administration, the tumors began to grow with a different time of appearance which was between 38-63 days. Since we used 6 biological replicates for each group based on Federer formula, 12 rats were used for IT and INT groups. Then, we selected the first 6 rats that developed tumors to be assigned to the IT group, so that it was technically easier to carry out the research and avoided mistakes in operating the ECCT device. We did not equally distribute the induced rats to both groups to avoid human errors in operating the ECCT, since we had to turn on and off the device twice every day for the IT group, while not for the INT group.

 3. The rats in the NIT group were normal rats with corn oil administration. They did not experience any DMBA administration.

 4. We used 3 nodules from 3 rats for each IT and INT group, since this sample size was sufficiently representative for the IHC method. We randomly selected the rats with nodules for histopathological analysis.

 5. Yes, we made a mistake for that sentence. We will correct it.
